# Epigenetic Regulation of Fatty Acid Amide Hydrolase in Alzheimer Disease

**DOI:** 10.1371/journal.pone.0039186

**Published:** 2012-06-12

**Authors:** Claudio D'Addario, Andrea Di Francesco, Beatrice Arosio, Cristina Gussago, Bernardo Dell'Osso, Monica Bari, Daniela Galimberti, Elio Scarpini, A. Carlo Altamura, Daniela Mari, Mauro Maccarrone

**Affiliations:** 1 Department of Biomedical Sciences, University of Teramo, Teramo, Italy; 2 Geriatric Unit, Department of Medical Sciences and Community Health, University of Milan, Fondazione Cà Granda, IRCCS Ospedale Maggiore Policlinico, Milan, Italy; 3 Department of Psychiatry, University of Milan, Fondazione Cà Granda, IRCCS Ospedale Maggiore Policlinico, Milan, Italy; 4 Department of Neurological Sciences, “Dino Ferrari” Center, University of Milan, Fondazione Cà Granda, IRCCS Ospedale Maggiore Policlinico, Milan, Italy; 5 Department of Experimental Medicine and Biochemical Sciences, Tor Vergata University, Rome, Italy; 6 European Center for Brain Research (CERC)/IRCCS Santa Lucia Foundation, Rome, Italy; University of Kentucky, United States of America

## Abstract

**Objective:**

Alzheimer disease (AD) is a progressive, degenerative and irreversible neurological disorder with few therapies available. In search for new potential targets, increasing evidence suggests a role for the endocannabinoid system (ECS) in the regulation of neurodegenerative processes.

**Methods:**

We have studied the gene expression status and the epigenetic regulation of ECS components in peripheral blood mononuclear cells (PBMCs) of subjects with late-onset AD (LOAD) and age-matched controls (CT).

**Results:**

We found an increase in fatty acid amide hydrolase (*faah*) gene expression in LOAD subjects (2.30±0.48) when compared to CT (1.00±0.14; *p<0.05) and no changes in the mRNA levels of any other gene of ECS elements. Consistently, we also observed in LOAD subjects an increase in FAAH protein levels (CT: 0.75±0.04; LOAD: 1.11±0.15; *p<0.05) and activity (pmol/min per mg protein CT: 103.80±8.73; LOAD: 125.10±4.00; *p<0.05), as well as a reduction in DNA methylation at *faah* gene promoter (CT: 55.90±4.60%; LOAD: 41.20±4.90%; *p<0.05).

**Conclusions:**

Present findings suggest the involvement of FAAH in the pathogenesis of AD, highlighting the importance of epigenetic mechanisms in enzyme regulation; they also point to FAAH as a new potential biomarker for AD in easily accessible peripheral cells.

## Introduction

Alzheimer disease (AD) is the most frequent form of dementia in the elderly, affecting more than 25 million people worldwide; it is characterized by progressive deterioration of cognition and memory as a result of selective neuronal loss in the hippocampus and cerebral cortex.

Current treatments for AD provide only palliative approaches [1], and in the search for new therapeutic targets the “endocannabinoid system” (ECS) recently emerged as a promising candidate, due to its role in neuroinflammatory and neurodegenerative diseases [2], [3]. In the past centuries, cannabinoids have been used for the treatment of various diseases [4], but only recently the mechanisms by which these compounds exert their effects began to be understood. G-protein-coupled type-1 and type-2 cannabinoid receptors (CB_1_ and CB_2_), located in both the central nervous system and the periphery, have been characterized along with their two main endogenous ligands, the ethanolamine of arachidonic acid [“anandamide” (AEA)], and 2-arachidonoyl-glycerol (2-AG) [4], [5]. AEA and 2-AG are endocannabinoids (eCBs) able to activate also non-CB_1_/non-CB_2_ receptors and/or a purported “CB_3_” (or GPR55) receptor. Furthermore AEA, but not 2-AG, behaves as a ligand to type-1 vanilloid receptor (TRPV1) channels (for a detailed review see Pertwee [6]). AEA is synthesized through multiple pathways, of which the best characterized is catalyzed by *N*-acyl-phosphatidylethanolamines-hydrolyzing phospholipase D (NAPE-PLD). The biological activity of AEA through its receptors is terminated upon intracellular degradation by fatty acid amide hydrolase (FAAH). Instead, 2-AG is mainly synthesized by an *sn*-1-specific diacylglycerol lipase (DAGL), and is degraded by a specific monoacylglycerol lipase (MAGL) (for detailed review see Di Marzo [7]). AEA, 2-AG and congeners, their target receptors and the respective metabolic enzymes form the ECS [5], [8], [9], [10].

Accumulated evidence shows that both exogenous plant-derived and endogenous cannabinoids are neuroprotective [11]. Additionally, the ECS is known to play a key role in pathological events occurring in the AD brain: excitotoxicity [12], oxidative stress [13] and inflammation [2]. Yet, few studies investigated alterations of the ECS in normal or pathological aging (reviewed in Paradisi et al. [14]). For example, it has been reported that CB_1_ receptors are decreased in aged rats [15], [16] and in human pathological conditions affecting basal ganglia structures [17], and CB_1_ receptor in human AD brains mainly demonstrates no changes [18], [19], [20] or decreased levels [21], [22].

CB_2_ is overexpressed on microglia within the senile plaque, and so is FAAH that in turn contributes to the inflammatory process of AD and to the release of pro-inflammatory molecules, such as arachidonic acid, from AEA and related eCBs [11]. Consistently with these results, an increase of both CB_2_ and FAAH expression was found in glial cells surrounding Aβ plaques in tissue samples from subjects with Down syndrome [23], a condition that shares common pathways with AD [24].

Moreover, in peripheral whole blood from AD subjects, an increase in CB_2_ gene expression was observed in patients with lower Mini Mental Status Exam (MMSE) scores [25]. In another study using peripheral markers, there were no differences in eCB concentrations in plasma samples from patients with AD and healthy controls. Moreover, circulating eCBs were not correlated with cognitive performance in subjects at risk of AD [26]. Overall, ECS gene expression and eCBs signaling in AD remain largely unexplored [27].

Our first aim was thus to evaluate possible changes of ECS gene transcription occurring in Late Onset (after age 65) AD (LOAD) subjects. We conducted the study on peripheral blood mononuclear cells (PBMCs), that are accessible cells with potential for biomarker discovery in neuroinflammatory disorders [2]. In this context, the study of gene regulation in blood cells from living patients offers the possibility to go through the whole history of the disorder (including response to pharmacological, metabolic and environmental events) in a more comprehensive perspective, compared to the single point postmortem brain studies.

Gene expression in the nervous system is modulated by epigenetic processes which consist of mitotically heritable, but reversible, changes in gene accessibility that occur without a change in the genomic DNA sequence [28], [29]. Main epigenetic mechanisms essentially include DNA methylation and histone modifications [30]. DNA methylation, in particular, consists of the transfer of a methyl group to position 5 of the cytosine pyrimidine ring of a cytosine guanine dinucleotide (CpG), which ultimately blocks the binding of transcription factors causing chromatin compaction and gene silencing [28].

Since epigenetic mechanisms have been also associated with a range of neurobiological processes in the brain, including central nervous system development, learning, memory and neurodegeneration [28], we investigated the role of DNA methylation in the regulation of ECS gene transcription in LOAD.

It is important to notice that PBMCs may be also a useful model of epigenetic gene regulation in the brain [31]. In fact, it has been shown that PBMCs share much of the non-synaptic biochemical environment of neurons and contain the full complement of epigenetic enzymes found in most tissues, including neurons and peripheral nucleated cells [32], [33].

## Methods

### Ethics statement

The study has been approved by the Institutional Review board of the Department of Internal Medicine, University of Milan, Fondazione Cà Granda, IRCCS Ospedale Maggiore Policlinico, Mangiagalli e Regina Elena. Informed written consent to participate in this study was given by patients and their caregivers.

### Subjects

The study involved LOAD patients (mean age ± standard deviation: 79.47±6.30 years – 23/9 females/males; n = 33 for DNA methylation studies and protein level detection, n = 13 for gene expression analysis) and non-demented age- and sex-matched healthy CT with similar educational levels (mean age ± standard deviation: 79.98±6.36 years; n = 33 for DNA methylation studies and protein level detection, n = 12 for gene expression analysis). All subjects were Caucasians living in Northern Italy, who were prospectively enrolled from a larger population of outpatients attending the Geriatric Unit of the Ospedale Maggiore IRCCS, University of Milan, Italy.

LOAD patients fulfilled the NINCDS-ADRDA criteria [34]. A computed tomography or magnetic resonance imaging scan corroborated the diagnosis of AD. Controls were carefully assessed in order to exclude the presence of neurological and cognitive disorders of any kind. Blood from all patients and controls was collected at the same time in the morning. At the recruitment, none of the subjects showed clinical signs of inflammation and were treated with acetylcholine esterase inhibitors.

All subjects and their relatives gave informed consent, and the study protocol was approved by the university hospital's Ethics Committee. The ApoE genotypes were determined as previously described [35].

All subjects underwent functional and cognitive evaluations. Basic activities of daily living (ADL; possible score: 0–6, with 6 being the best score) and instrumental ADLs (IADL; possible score: 0–8, with 8 being the best score) were assessed, as well as cognitive measures obtained using the Mini Mental Status Exam (MMSE) [36], [25], a widely used dementia severity test with scores ranging from 0 to 30 points. Table 1 summarizes the detailed pathological informations about CT and LOAD subjects.

**Table 1:** Description of study sample.

*Test*	CT	LOAD	p values (Mann-Whitney U test)
MMSE	28.37±0.22	17.65±1.39	*<0.0001*
ADL	5.15±0.23	3.63±0.29	*0.0006*

*ApoE genotype*	CT	LOAD	p value (X2 test)
ε4 carriers (%)	17	43	*0.032*
ε4 non carriers (%)	83	57	

Data are represented as means ± SEM. Mini-Mental-State Examination (MMSE) normal range: 0–30. Activities of Daily Living (ADL) normal range: 0–6.

### Analysis of gene expression by quantitative real-time reverse transcription–polymerase chain reaction (qRT-PCR)

PBMCs were separated by density gradient using the Lympholyte-H kit (Cedarlane Laboratories Limited, Canada), and total RNA was isolated using standard procedures [37]. Relative abundance of each mRNA species was assessed by real-time qRT-PCR, using iQ SYBR Green Supermix (Bio-Rad, Hercules, CA, USA) on an DNA Engine Opticon 2 Continuous Fluorescence Detection System (MJ Research, Waltham, MA, USA). All data were normalized to the endogenous reference gene glyceraldehyde-3-phosphate dehydrogenase (GAPDH). Differences in threshold cycle (Ct) number were used to quantify the relative amount of PCR target contained in each tube. Relative expression of different gene transcripts was calculated by the Delta-Delta Ct (DDCt) method and converted to relative expression ratio (2^−DDCt^) for statistical analysis. After PCR, a dissociation melting curve was constructed in the range of 60°C to 95°C to evaluate the specificity of the amplification products. The primers used for PCR amplification, designed using Primer 3, are shown in table 2.

**Table 2:** Primers used for gene expression and DNA methylation analysis.

Human Gene	Forward	Reverse
CB_1_	CCTTTTGCTGCCTAAATCCAC	CCACTGCTCAAACATCTGAC
CB_2_	TCAACCCTGTCATCTATGCTC	AGTCAGTCCCAACACTCATC
“CB_3_”	ATCTACATGATCAACCTGGC	ATGAAGCAGATGGTGAAGACGC
TRPV1	TCACCTACATCCTCCTGCTC	AAGTTCTTCCAGTGTCTGCC
NAPE-PLD	TTGTGAATCCGTGGCCAACATGG	TACTGCGATGGTGAAGCACG
FAAH	CCCAATGGCTTAAAGGACTG	ATGAACCGCAGACACAAC
DAGL	GCCACCAAGAGGAGGCAGCG	CCGCGTGCAGCAGAGGAACA
MAGL	ATGCAGAAAGACTACCCTGGGC	TTATTCCGAGAGAGCACGC
GAPDH	GATTCCACCCATGGCAAATTC	TGGGATTTCCATTGATGACAAG
M_FAAH	CGTTTTGGTTTGTTGTTTCGT	CTATCCATATTCTCCAAACCCG
U_FAAH	GTTGTTTTGGTTTGTTGTTTTGT	CTATCCATATTCTCCAAACCCACT
myoD	CCAACTCCAAATCCCCTCTCTAT	TGATTAATTTAGATTGGGTTTAGAGAAGGA

### Analysis of DNA methylation by methylation-specific primer real-time PCR

Genomic DNA was extracted from blood using a salting-out method [38], and was subjected to bisulphite modification using a commercially available modification Kit (Zymo Research, Irvine, CA, USA). Methylation analysis was performed by fluorescence-based real-time PCR. Amplified *faah* sequence contained 18 CpG sites (M_FAAH), and was located within exon 1 of the gene. It has recently been shown that DNA methylation downstream of the transcription start site, in the region of the first exon, is much more tightly linked to transcriptional silencing than is methylation in the upstream promoter region [39]. PCR was also performed for non-CpG-containing region of myoD, that served as control gene. Bisulfite-modified CpGenome™ universal unmethylated DNA (Chemicon International, Temecula CA, USA) was used as negative control. The percentage of methylation was calculated by the 2^−DDCt^ method, where DDCt = (Ct, M_FAAH – Ct, myoD) sample – (Ct, M_FAAH – Ct, myoD) fully methylated DNA, multiplying by 100. For relative quantifications, standard curves were generated separately for each gene and myoD from serial dilutions of bisulfite modified CpGenome™ universal methylated DNA (Chemicon International, Temecula CA, USA). To confirm our results, we also used primers for the unmethylated DNA sequence (U_FAAH) in selected DNA bisulfite-converted samples calculating the % of methylation as previously reported [40]–[41]. These latter data are not shown to avoid redundancy. The primers are shown in table 2.

### Analysis of protein levels by Western blotting

PBMCs were disrupted by lysis buffer containing 2% SDS and 0.1% protease inhibitor cocktail (Sigma-Aldrich, St. Louis, MO, USA). Equal amounts of total extracts (30 µg of protein) were electrophoresed on 10% acrylamide gels and transferred to PVDF membranes (Amersham Biosciences, Psicataway, NJ, USA). Membranes were saturated with a solution of 5% non-fat dry milk, then they were probed with goat anti-FAAH polyclonal antibody (Santa Cruz Biotechnology Inc, Santa Cruz, CA, USA) at 1∶250 dilution. Secondary antibody against goat IgG (Santa Cruz Biotechnology Inc.) was used at 1∶1000. The antigen-antibody complex was detected by enhanced chemiluminescence reagents (ECL, Amersham Biosciences), quantifying the intensities of the immunoreactive bands by densitometric analysis through the ImageJ software (NIH, Bethesda, Maryland, USA). The specificity of the anti-FAAH antibody was checked by using mouse liver extracts as positive control [42], and human HeLa cell lysates as negative control [43], under the same experimental conditions. Equal loading was further ascertained by re-probing membranes with a mouse anti-β-actin monoclonal antibody, diluted 1∶10000 (Cell Signaling, Billerica, MA, USA).

### Assay of FAAH activity

The hydrolysis of 2.5 µM ^3^H-AEA (200 Ci/mmol, from Perkin Elmer, MA, USA) by FAAH (E.C. 3.5.1.4) was assayed in PBMC extracts (20 µg protein per test) by reversed-phase high performance liquid chromatography, as reported [44]. FAAH activity was expressed as pmol ^3^H-arachidonic acid released from ^3^H-AEA per min per mg protein.

### Statistical analysis

All results are expressed as mean ± standard error of the mean (SEM). Statistical differences of *faah* gene expression, DNA methylation changes at *faah* promoter as well as FAAH protein levels and enzyme activity of LOAD patients *versus* control subjects were calculated by the non-parametric Mann-Whitney U test through the Prism 5 program (GraphPad Software Inc., San Diego, CA). The evaluation of DNA methylation changes at *faah* promoter in relation to MMSE scores was analyzed by one-way ANOVA, followed by Newman-Keuls Multiple Comparison Test. To examine if a correlation existed between DNA methylation and MMSE score or gene expression changes, non-parametric Spearman or Pearson correlation analysis was performed. p values<0.05 were considered statistically significant.

## Results

**Figure 1:**
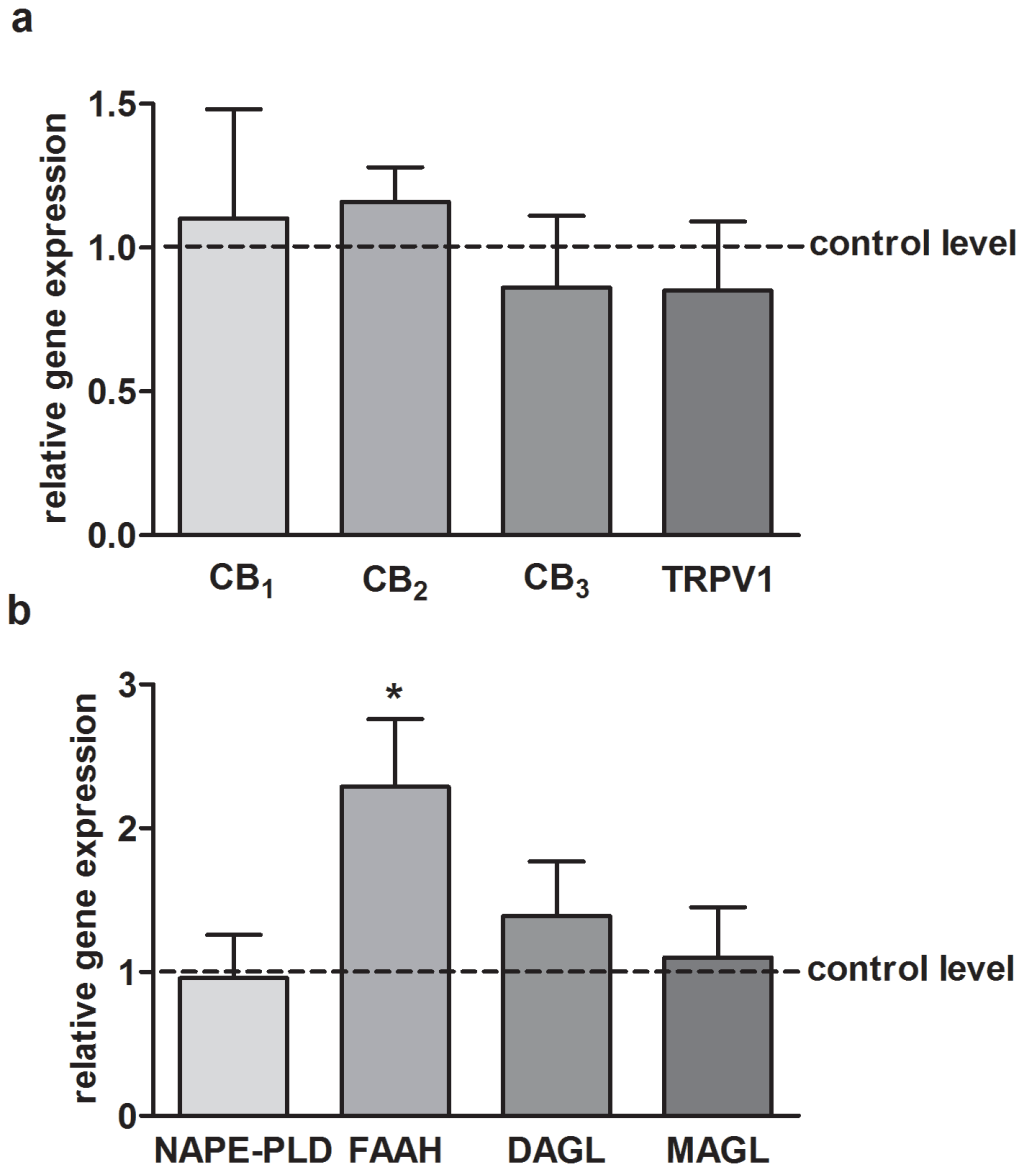
ECS gene expression levels. ECS genes (panel a: receptors; panel b: metabolic enzymes) expression in PBMCs from LOAD subjects. Bars represent 2^−DDCt^ values calculated by Delta–Delta Ct (DDCt) method. Expression was normalized to GAPDH, and data are represented as means ± SEM. *p<0.05 *vs* control.

**Figure 2:**
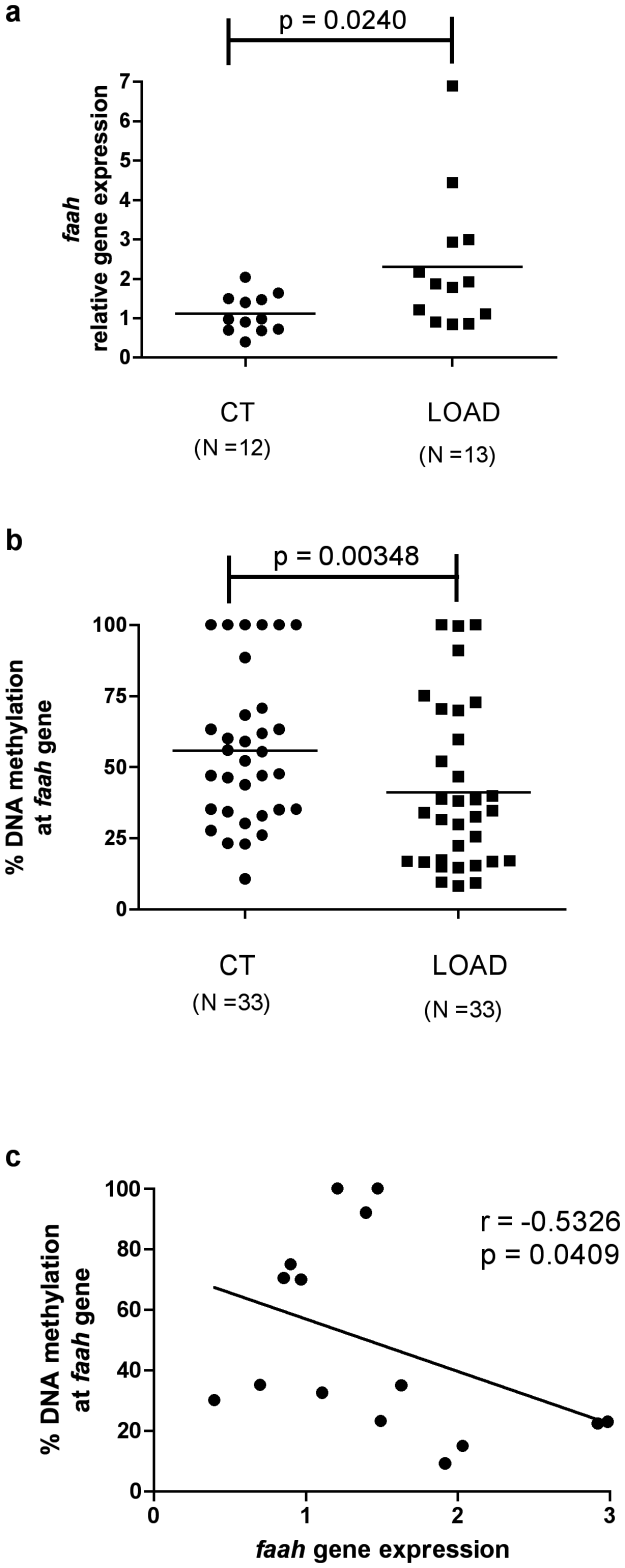
Genetic and epigenetic regulation of *faah* gene. a: Levels of FAAH mRNA in PBMCs from LOAD patients (n = 13) and controls (n = 12); b: Amount of methylated DNA at *faah* gene in controls (n = 33 ) and LOAD subjects (n = 33); c: Correlation between *faah* gene expression and % change of DNA methylation in LOAD subjects. Data were compared by Spearman's rank correlation coefficient (p<0.05, r = −0.5326). Scatter dots represent 2^−DDCt^ values calculated by Delta-Delta Ct (DDCt) method, as described in the Materials and Methods section.

Gene expression of all major ECS elements in LOAD subjects and healthy controls was quantified and only FAAH mRNA was found to be altered by the disease (Figure 1). A significant increase of *faah* gene expression was observed in LOAD subjects compared to controls (LOAD: 2.30±0.48; CT: 1.00±0.14;* p<0.05) (Figure 2a). Also a decreased DNA methylation at *faah* gene was observed in LOAD patients compared to controls (CT: 55.90±4.60%, LOAD: 41.20±4.90%; * p<0.05) (Figure 2b). Consistently, a significant inverse correlation between gene expression and DNA methylation levels (Spearman r = −0.5326, * p<0.05) was evident in all samples that were large enough to allow both assays (Figure 2c).

**Figure 3:**
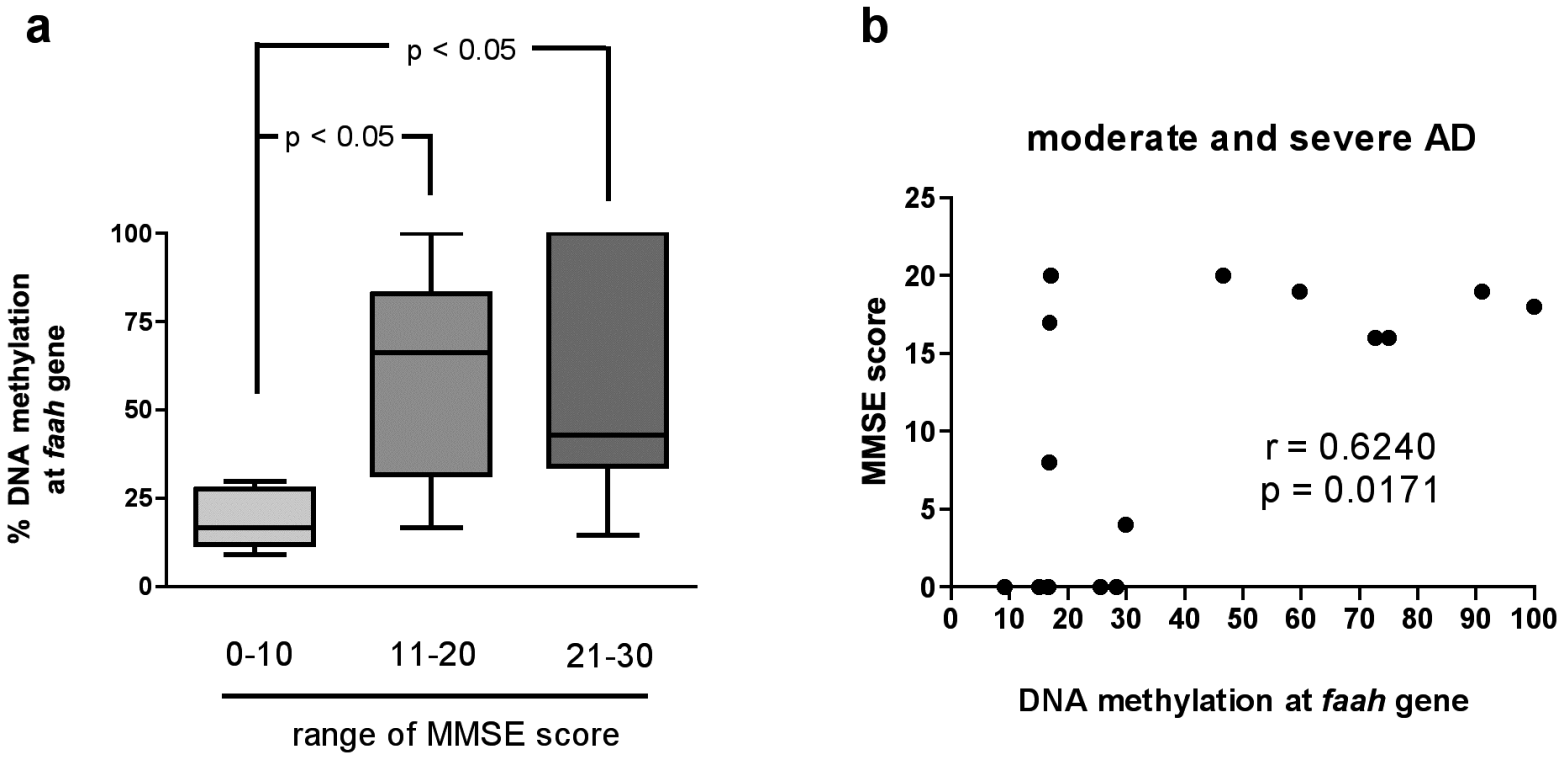
*faah* DNA methylation levels *vs* MMSE score. a: Amount of methylated DNA at *faah* gene in LOAD subjects subgrouped on the basis of MMSE score; b: Correlation between changes in DNA methylation at *faah* gene and LOAD subjects with severe AD, based on MMSE score. Data were compared by Pearson's rank correlation coefficient (p<0.05, r = −0.6240).

When data were stratified according to functional and cognitive tests, a significantly lower level of DNA methylation at *faah* gene was observed in LOAD subjects with lower levels of MMSE, and severe AD (score range 0–10 (n = 7): 19±3%; * p<0.05 Newman-Keuls) compared to the rest of the samples, moderate AD (score range 11–20 (n = 8): 59±11%) and incipient AD and/or controls (score range 21–30 (n = 24): 50±6%) (ANOVA: p = 0.0396; F = 3.545) (Figure 3a). Moreover, within subjects with moderate and severe AD, it was also observed a significant correlation with DNA methylation levels (Pearson r = 0.6240, *p<0.05) (Figure 3b).

**Figure 4:**
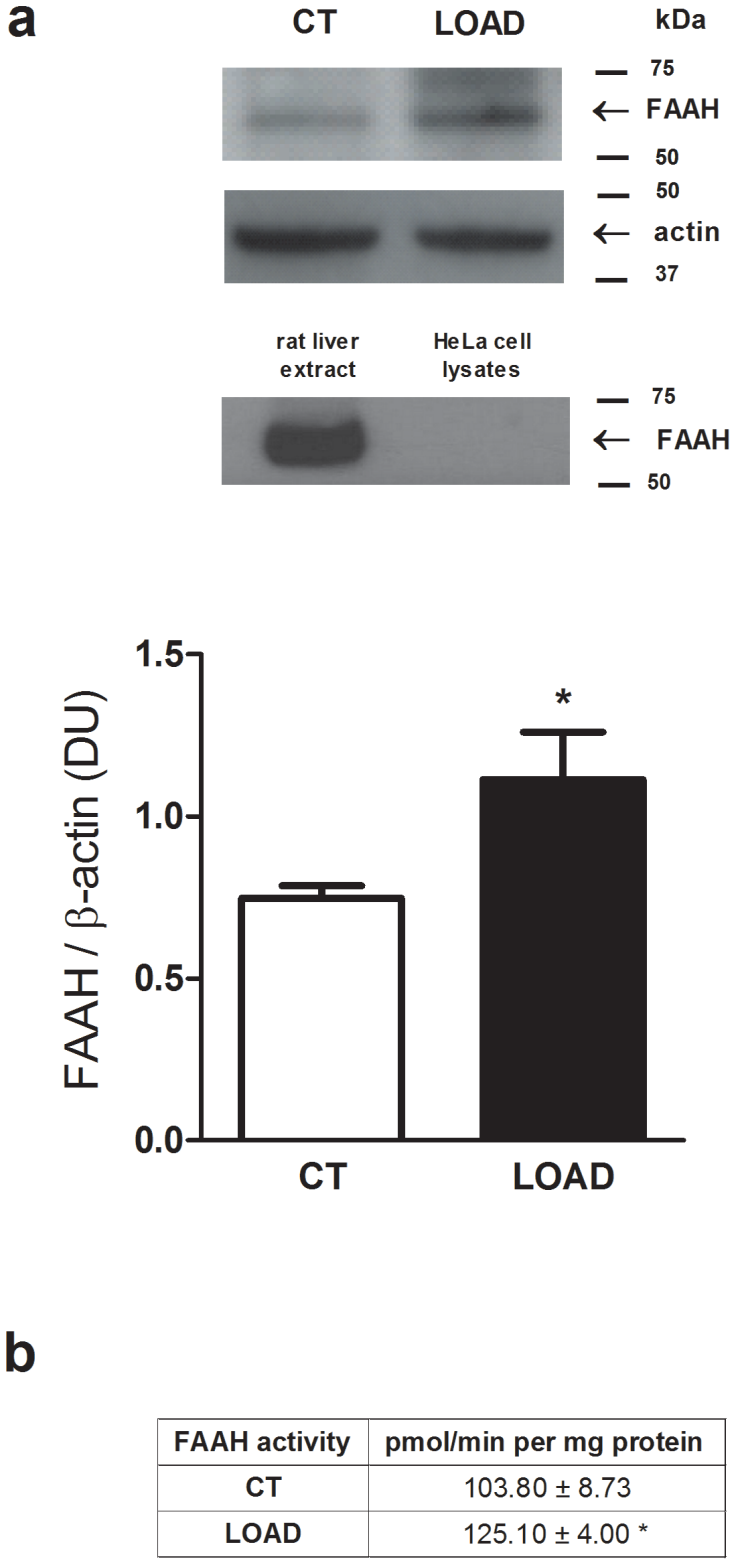
Levels of FAAH protein and activity. a: Analysis of FAAH protein levels in PBMCs from LOAD and control (CT) subjects. Values represent means ± SEM, *p<0.05 *vs* CT. Representative immunoblots of PBMC lysates reacted with specific anti-FAAH or anti-actin antibodies are shown, as well as FAAH immunoreactivity in rat liver extracts and HeLa cell lysates, used as positive and negative controls respectively. Molecular mass markers and the positions of FAAH and actin are indicated to the right. b: FAAH activity in LOAD and CT subjects, expressed as pmol/min per mg of protein (means ± SEM).

Protein samples from 28/32 CT/LOAD subjects were randomly pooled in five groups for FAAH protein detection. Western blot analysis revealed an increase in the levels of FAAH protein in LOAD subject compared to controls (1.11±0.15; 0.75±0.04; *p<0.05, n = 5) (Figure 4a). A significant difference was also evident when comparing FAAH activity between CT (103.80±8.73 pmol/min per mg protein) and LOAD (125.10±4.00 pmol/min per mg protein; *p<0.05) (Figure 4b).

## Discussion

Many studies have recently documented a role for ECS in several neurological diseases, among which AD is a new promising area of research [27], [45].

Inflammatory processes are closely associated to the neuropathological and cognitive syndromes of AD [46], and the anti-inflammatory actions of ECS may allow their exploitation for the treatment of the disease [47].

Here, we investigated possible changes in gene expression of all major ECS elements in PBMCs of LOAD subjects compared to healthy controls, and we observed a remarkable up-regulation of the *faah* gene in AD subjects without changes in any other ECS gene (Figure 1). FAAH is one of the enzymes involved in the termination of endocannabinoid signaling, and it is able to hydrolyze AEA and other fatty acid amides. FAAH protein is abundantly expressed in large neurons [48], as well as in white matter astrocytes [49]. *In vivo* and *in vitro* data indicate that FAAH inhibition could be a promising therapeutic strategy against excitotoxic damage.

We thus confirmed in human peripheral cells the *faah* mRNA up-regulation already observed in AD postmortem brains [18]. Unfortunately, due to methodological constraints, it was not possible to isolate PBMC for RNA extraction from all patients recruited. Others have mainly focused the attention on the mRNA expression of CB_1_ receptors, the most abundant G-protein-coupled receptor within the brain, although with conflicting results [19], [21], [22]. To the best of our knowledge this is the first study on the expression of all the elements of the ECS in PBMCs of LOAD subjects, where *faah* gene clearly appears to be the only component of the system that is altered in the disease.

These intriguing results prompted us to investigate in more detail the regulation of FAAH expression in AD. Our aim was not only to evaluate changes in mRNA, protein and activity levels, but also to study the epigenetic regulation of *faah* gene transcription.

Indeed we report that FAAH protein, and hence enzyme activity, increased in PBMCs of LOAD subjects (Figure 4a–b). Again, this finding in peripheral cells is in agreement with previous results in postmortem AD brains [18], where FAAH protein up-regulation within plaques was suggested to lead to an increase in metabolites from AEA degradation (such as arachidonic acid). Such metabolites could contribute to the inflammatory process occurring in AD. This hypothesis is also in line with the neuroprotective effect observed upon CB_1_ receptor stimulation [12], and with the role that ECS might have in pathological processes like amyloid protein accumulation in AD [3]. In this context, it should be noted that it has been recently reported that circulating eCBs levels do not correlate with cognitive performance in subjects at risk of AD [26]. However, the endogenous tone of AEA is controlled by multiple biosynthetic and hydrolytic pathways [7], therefore it is not unprecedented that a difference in FAAH activity and expression is not reflected by a different content of AEA. Moreover, blood cells other than PBMCs are known to actively metabolize AEA, e.g. platelets [50], thus FAAH of PBMCs is only one of the contributors to the control of circulating AEA concentrations. At any rate, our data clearly identify FAAH from PBMCs as a potential biomarker of AD.

Interestingly, our present findings speak in favor of the relevance of PBMCs as peripheral markers that could mirror the pathology within the brain. In most of the illnesses, it is possible to study the affected tissue while the patient is still alive, yet this option is rather difficult in subjects with neurodegenerative disorders. Previous studies already suggested that PBMCs are a more accessible source of biomarkers in psychiatric [31] and neurodegenerative/neuroinflammatory disorders [2]. We thus studied DNA methylation at *faah* gene in PBMCs, since epigenetic mechanisms represent a link between gene expression alterations and environmental factors. In line with this, we and others have suggested a potential role for epigenetic effects in the development of AD. For instance, recent data from our group showed a significant increase in Pin1 gene expression together with a significant decrease in gene promoter methylation in PBMCs of LOAD patients [51]. It has also been observed that AD patients display high homocysteine and low B12 vitamin and folate in blood, suggesting a dysregulation in the *S*-adenosylmethionine cycle that contributes methyl donors for DNA methylation [52]. Moreover, an unusual methylation pattern occurring with age in LOAD subjects has been identified [53]. Here, we found a correlation between the increase of FAAH mRNA and the reduction of DNA methylation in LOAD subjects. Moreover, in those patients with the most severe cognitive impairment we observed the lowest levels of the epigenetic mark.

Again, this is the first report showing a link between selective *faah* gene expression alteration and DNA methylation in LOAD. The reversibility of epigenetic marks might be of particular clinical importance to elucidate the action of existing pharmacological treatments, as well as to develop new therapeutic tools.

Epidemiologic and laboratory studies suggest the use of non-steroidal anti-inflammatory drug (NSAIDs), which act as non-selective inhibitors of cyclooxygenases (COXs), for the treatment of AD, based on their effect in reducing the inflammation surrounding amyloid plaques [54]. AEA is also a substrate of COX-2, and it has been suggested that its oxygenation serves as a mechanism to terminate eCBs signaling [55]. This is of particular interest in the central nervous system, where COX-2 inhibitors might achieve therapeutic effects in AD by increasing eCBs levels. However, due to the well-known side effects of NSAIDs, our data could give a more realistic alternative for AD treatment. In fact, it is well-known that AEA protects brain from inflammation and, based on our observations, we could speculate that inhibitors of FAAH activity could be more beneficial than NSAIDs in preventing the inflammatory process associated with Aβ deposition.

In conclusion, this study adds new information on ECS alterations in normal or pathological aging and, on the basis of the relationship between the brain and the periphery in AD, it also suggests a possible role for FAAH not only as a peripheral biomarker but also as new possible therapeutic target for AD.
